# Exploring the Use of Storytelling in Vaccination: A Scoping Review

**DOI:** 10.3390/vaccines14080665

**Published:** 2026-07-30

**Authors:** Marthe Bogne Penka, Johanna C. Meyer, Lubayna Khan, Tshepiso Mbangiwa, Benjamin M. Kagina, Rudzani Muloiwa, Ruth Stewart

**Affiliations:** 1 Vaccines for Africa Initiative and NITAG Support Hub (NISH), Faculty of Health Sciences, University of Cape Town, Observatory, Cape Town 7925, South Africa; khnlub002@myuct.ac.za (L.K.); tshepiso.mbangiwa@uct.ac.za (T.M.); benjamin.kagina@uct.ac.za (B.M.K.); rudzani.muloiwa@uct.ac.za (R.M.); ruth@futureevidence.org (R.S.); 2 Department of Paediatrics and Child Health, Faculty of Health Sciences, University of Cape Town, Rondebosch, Cape Town 7700, South Africa; 3 School of Public Health, Faculty of Health Sciences, University of Cape Town, Observatory, Cape Town 7925, South Africa; 4 Department of Public Health Pharmacy and Management, School of Pharmacy, Sefako Makgatho Health Sciences University, Garankuwa, Pretoria 0208, South Africa; 5 South African Vaccination and Immunisation Centre, Sefako Makgatho Health Sciences University, Garankuwa, Pretoria 0208, South Africa; 6 University College London, Gower Street, London WC1E 6BT, UK; 7 Future Evidence Foundation, Level 5, 485 Latrobe Street, Melbourne, VIC 3000, Australia

**Keywords:** communication, storytelling, vaccination, immunization

## Abstract

**Introduction:** Storytelling is gaining interest as a potentially useful research and communication approach in health. However, there remains a huge gap in synthesized evidence in relation to its use in vaccination. We set out to map existing evidence on the use of storytelling in vaccination practice. **Methods:** This review was conducted in accordance with Joanna Briggs Institute methodology and PRISMA-ScR guidelines. Databases were searched for eligible studies using search strings in PubMed, Scopus, Academic Search Premier, Africa Wide Information, PsycINFO, ERIC, Cochrane Library, Web of Science Core Collection, and SciELO Citation Index via Web of Science. The gray literature was searched on Primo and Google Scholar. References from other sources were identified through a manual search using Google. Identified citations were uploaded into Covidence for screening and data extraction. Qualitative content analysis was used to summarize data in conceptual categories, including frequency counts. **Results:** Overall, 6236 studies were identified and imported into Covidence. Following removal of duplicates, 3804 studies were screened for title and abstract. 386 studies met eligibility criteria for full-text screening, which led to 110 included studies. Storytelling was used in immunization between 1997 and 2025 as a communication intervention (96/110 studies), research method (12/110 studies), and therapeutic approach (3/110 studies). Most studies originated from North America (67/110 studies), while six originated from Africa. Two-thirds of included studies (64/110 studies) were randomized controlled trials. Among the 62 studies reporting vaccine-related outcomes, 80.6% (50/62 studies) reported changes in one or more vaccine-related outcomes following exposure to storytelling interventions. The trustworthiness of these outcome findings is unknown. Reported changes were more frequently observed for cognitive, affective, and intentional outcomes than for behavioral outcomes such as vaccine uptake. Again, these findings have not been subject to critical appraisal. **Conclusions:** Storytelling is emerging as an adaptable approach with potential for addressing contextual vaccine challenges. Although several studies reported changes in cognitive, affective, and intentional outcomes, this scoping review cannot ascertain whether these changes were attributed to storytelling itself.

## 1. Introduction

Vaccine uptake continues to be threatened by the ongoing infodemic crisis, which has undermined global immunization hard-won gains and progress [1]. The widespread circulation of inaccurate and misleading information, especially on social media, has negatively influenced vaccine literacy and confidence, contributing to vaccine hesitancy, which WHO identifies as a major threat to global health [2,3]. The progress made over the years in global immunization—estimated to have saved 154 million lives between 1974 and 2024—has stalled [4]. This includes impeded progress towards global disease elimination and eradication, a risk of 1.6 million avoidable future deaths from vaccine-preventable diseases, large-scale and disruptive outbreaks, and a persistently high number of zero-dose and under-immunized children [4,5]. There is a sustained global concern regarding addressing vaccine hesitancy to restore high and equitable immunization coverage [6,7,8,9].

Vaccine hesitancy is defined as the delay in acceptance or refusal to take vaccines despite the availability of vaccination services [10]. This is a behavioral phenomenon of indecisiveness about vaccination. The hesitant population may accept a vaccine though unsure, may accept only some vaccines, or delay or refuse some, or refuse but remain unsure [11]. Vaccine hesitancy is highly dynamic and varies by geography, time, vaccine type, and individuals. As a complex and context-specific issue, it requires tailored diagnosis and context-appropriate interventions for effective response [12,13]. The WHO SAGE working group developed a 3C vaccine hesitancy model to map factors influencing vaccine uptake [11]. This model demonstrates that vaccine hesitancy is influenced by both trust and other complex factors. These include three key determinants—confidence, complacency, and convenience—which may act as barriers to vaccine uptake. Betsch and colleagues extended the 3C model to a 5C scale for the measurement of the psychological antecedents of vaccine acceptance. The 5C model suggests that, beyond confidence, complacency, and convenience, other psychological factors greatly influence vaccine uptake, which in addition are calculation and collective responsibility [14].

Assessing vaccine hesitancy requires contextualizing individual and group influences and vaccine-specific related issues [15]. Contextual factors associated with vaccine hesitancy include socio-economic, cultural, religious, geographical, access, social approval, communication, and media [16]. Individual factors entail education level, knowledge regarding the vaccine, risk perception, health beliefs, and experiences with healthcare services [17]. Vaccine-specific issues include vaccination schedules, the way new vaccines are introduced, and the strength of vaccine recommendations [18].

To build vaccine confidence, WHO recommends dialog-based interventions that directly engage unvaccinated and under-vaccinated communities and address the underlying determinants of vaccine hesitancy and low vaccine uptake. Well-tailored persuasive communication interventions, community engagement interventions, and digital interventions are some recommended approaches to enhancing vaccine confidence [11,19]. Storytelling provides a potential solution as it is known to be engaging and persuasive in shaping people’s choices [20,21,22,23,24].

Storytelling is known as the act of presenting a story with causal links between events, engaging the imagination of the audience through a vivid presentation of ideas, beliefs, personal experiences, life lessons, or research evidence while evoking powerful emotions and insights to persuade change, entertain, share knowledge/educate, and preserve culture [25,26,27]. Storytelling can be either entertaining or serious. The former is delivered solely to provide amusement or enjoyment, while serious storytelling goes beyond entertainment and results from a thoughtful process designed to help improve individuals’ attitudes, knowledge, and beliefs or to inform policy [28] and practice [29].

The Cambridge Dictionary defines a story as a description, either true or imagined, of a connected series of events. Neuroscientists are uncovering evidence of the impact of storytelling on the listener’s brain, in engaging their imagination and emotions, facilitating retention, persuasion, understanding, and behavior change [30,31,32]. Storytelling has been used globally, including in Africa, as a tool to educate, engage, inspire, and influence change [33,34]. Its relationship to misinformation suggests that it can be used as a force for good, as well as a tool for spreading confusion and falsehoods [35]. Despite the potential of storytelling as a research and health communication tool, and its frequent use in spreading misinformation, little is known about how storytelling is used in the field of vaccination. We therefore aimed to explore the use of storytelling in vaccination, including the different instances and approaches applied, and vaccine-related outcomes achieved.

A preliminary search conducted on PubMed and the Cochrane Library could not identify any current or underway systematic reviews or scoping reviews on the topic. However, a search of the JBI Evidence Synthesis library identified two relevant title registrations. One had a much broader scope with no focus on vaccination [36], whereas the other was an effectiveness review that fell outside the scope of this review [37]. Whilst the findings of these two reviews will be of great interest, they will not be duplicative of the current review. This review makes a significant contribution to the literature by synthesizing current research on storytelling and its application in the field of immunization. Additionally, this review offers a preliminary basis for an effectiveness review.

## 2. Materials and Methods

The current scoping review was conducted in accordance with the Joanna Briggs Institute (JBI) methodology for scoping reviews [38].

### 2.1. Review Questions

**Main review question:** In what ways has storytelling been used in the field of vaccination?

**Review sub-questions:**

1. What are the different formats and channels used for storytelling in the field of vaccination?
2. What kind of evidence is available on storytelling as a communication intervention for vaccination?
3. Which outcomes have been considered in research about the influence of storytelling on vaccination?

### 2.2. Inclusion Criteria

#### 2.2.1. Participants

We were interested in the following participants: the public at large, including citizens (for the purpose of this review, a citizen is considered a member of a community who, in principle, is in a position to help shape the shared life, direction, and practices of that community) [39], health practitioners, health researchers, and health policy makers.

#### 2.2.2. Concept

We included all studies targeting “serious” storytelling in the field of vaccination. Serious storytelling is here defined as stories that go beyond merely entertaining but serve to educate or generate knowledge as identified through the objectives and outcomes reported in the included studies. Additionally, eligible studies had to deliberately employ narrative structure and core story elements, including a setting, plot, character(s), narrative style, and theme (which in this review was vaccination).

Thus, standalone testimonial messages which were not part of an empirical research study were excluded. We also excluded qualitative interviews to gather lived experiences that did not intentionally employ a storytelling or narrative inquiry approach and did not incorporate identifiable story elements and narrative structure. Furthermore, studies reporting on entertaining storytelling with no direct link to vaccination were excluded. We excluded standalone audiovisual educational materials that were not used within empirical studies as a storytelling intervention. Studies on storytelling related to health issues other than vaccination were also excluded. All studies using stories or narratives solely as a reporting strategy, e.g., narrative review, vaccine success story, and not as a core component of the research objective, methodology, intervention, and outcome were excluded. We also excluded all studies using storytelling terms solely as opinion pieces or discourse with no application of storytelling as a research method or intervention, e.g., “the success story of vaccines”.

#### 2.2.3. Context

This review had no geographical limitation and explored storytelling in the context of vaccination globally. In this review, the terms “vaccination” and “immunization” were used interchangeably to refer to the same concept, reflecting their often-synonymous use in the literature. Similarly, “storytelling” and “narrative” were used interchangeably in line with their frequent overlapping use in the literature.

#### 2.2.4. Types of Sources

This scoping review considered both experimental and quasi-experimental study designs, including randomized controlled trials, non-randomized controlled trials, before and after studies and interrupted time-series studies. In addition, analytical observational studies including prospective and retrospective cohort studies, case–control studies and analytical cross-sectional studies, descriptive observational study designs including case series, individual case reports, descriptive cross-sectional studies, qualitative and mixed-method studies were considered for inclusion in the review. Reviews, editorials, and opinion papers were excluded from the synthesis. However, interesting reviews were screened to identify additional primary studies that met the inclusion criteria not captured through our initial search.

### 2.3. Search Strategy

The search strategy (Refer to Supplementary File S1) aimed to locate both published and unpublished studies. A three-step search strategy was utilized in this review. First, an initial limited search of MEDLINE (PubMed) and ProQuest was undertaken to identify articles on the topic. The text words contained in the titles and abstracts of relevant articles, and the index terms used to describe the articles, were used to develop a full search. The search strategy was reviewed by a librarian and senior authors. The search strategy included all identified keywords and index terms adapted for each included database and/or information source (Refer to Supplementary File S2). We searched PubMed, Scopus (including EMBASE), Academic Search Premier, AfricaWide Information, PsycINFO and ERIC via EBSCOhost, Cochrane Library, Web of Science Core Collection and SciELO Citation Index (Scientific Electronic Library Online) via Web of Science, to identify eligible studies for inclusion. We developed search strings for each database. A gray literature search was conducted to identify studies not indexed in the above databases. The gray literature search was conducted on Primo and Google Scholar. References from other sources were identified through a manual search using Google. The search was done with no language restriction; however, all identified records were in English.

### 2.4. Study/Source of Evidence Selection

All identified citations were collected and uploaded to Covidence [40], a web-based data extraction platform. After the removal of duplicates, two reviewers independently screened the titles, abstracts, and full text of the identified records for assessment against the inclusion criteria for this review. Full texts not meeting the inclusion criteria were excluded, with reasons for exclusion provided. Disagreements were resolved through discussions or by involving a third reviewer. Management of studies was done using Covidence, and a PRISMA flow diagram was generated through Covidence to demonstrate the search results and process.

### 2.5. Data Extraction

A data extraction form was prepared and customized within the Covidence platform, based on the review inclusion criteria. The data extraction form was piloted before the commencement of the review (Refer to Supplementary File S3). Two reviewers independently extracted the data. All disagreements were resolved by discussion or by involving a third reviewer. The following data were recorded for each study:

- Author;
- Year of publication;
- Origin (where the publication was published);
- Aim of the publication;
- Sample characteristics (setting, gender, age, number of participants, socioeconomic status);
- Methodology within the study;
- Intervention type and its comparator (details; duration of the intervention, follow-up, etc.);
- Outcome and how it was measured; and
- Key findings relevant to the review.

### 2.6. Assessment of Bias

We explored the need to assess potential bias within the included studies using established checklists and risk-of-bias tools suitable for the methods employed within the studies. Including tools such as the JBI checklists for observational studies, the Critical Appraisal Skills Programme qualitative checklist [37] and the Cochrane Risk of Bias tool (RoB 2) [38]. However, given the focus of the review on the characteristics of the research and their use of storytelling, and not on the findings of the included studies, this was deemed unnecessary.

### 2.7. Data Analysis and Presentation

The analysis focused on summarizing and synthesizing evidence from within the literature on how storytelling is used in vaccination and its associated vaccine-related outcomes. Data have been summarized based on frequency counts and qualitative content analysis. The analysis included a systematic map of the extracted data, presented using tables and diagrams accompanied by descriptive summaries.

Results have been disaggregated under different conceptual categories. The scoping review reporting has been done using the JBI methodology and complemented by the PRISMA-ScR reporting checklist [39,40].

## 3. Results

**Figure 1:**
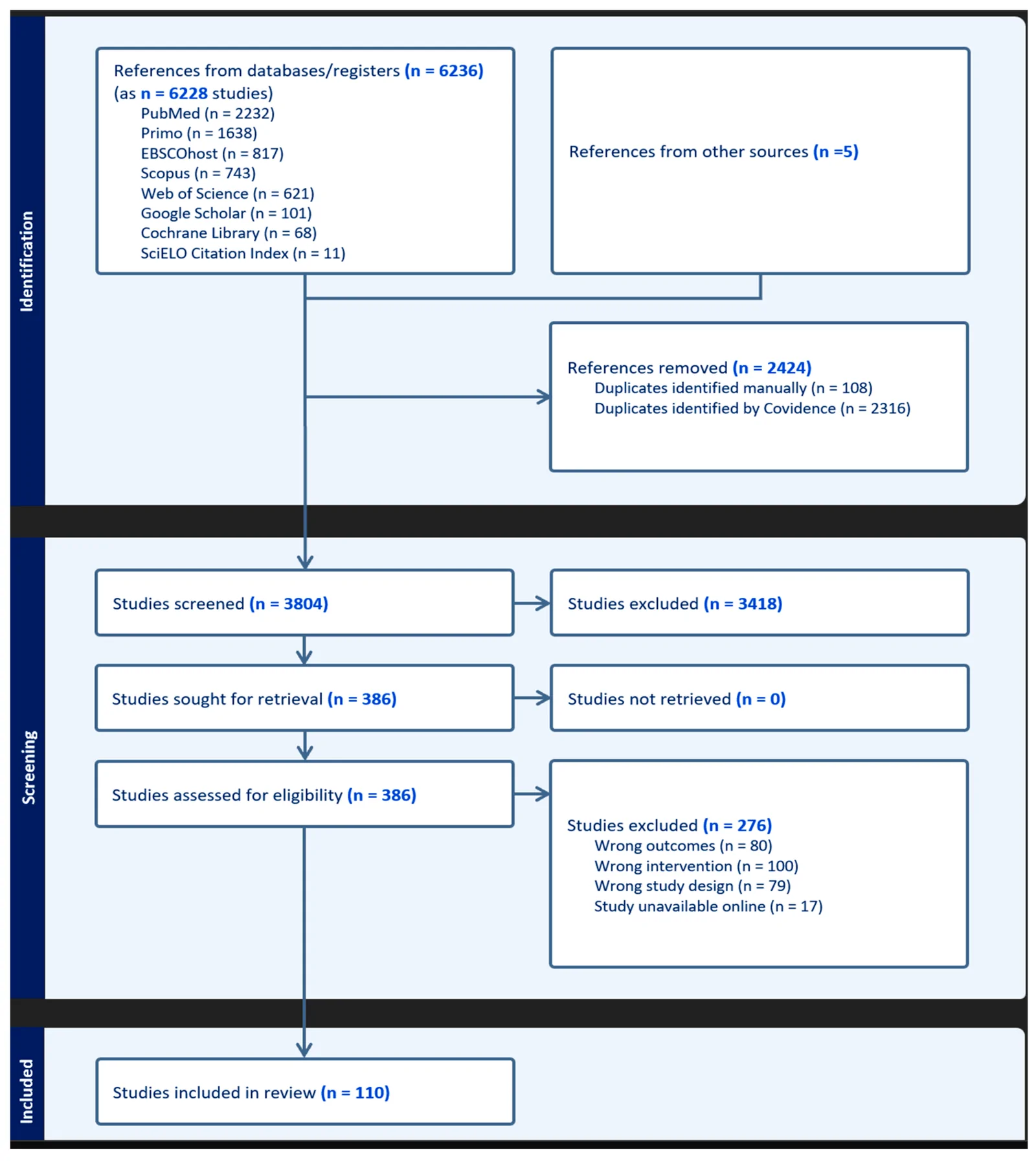
PRISMA-ScR flowchart of retrieved articles, showing the 110 studies included in the final analysis.

A total of 6236 studies were identified and imported into Covidence. After removal of duplicates, 3804 studies underwent title and abstract screening, of which 386 met the eligibility criteria and were considered for full-text screening. Finally, a total of 110 studies met the inclusion criteria and were included in this review (see Figure 1). We did not intentionally restrict inclusion to English-language studies. However, the final list included only English-language studies.

### 3.1. Characteristics of Included Studies

#### 3.1.1. Study Designs Reported in the Included Studies

**Figure 2:**
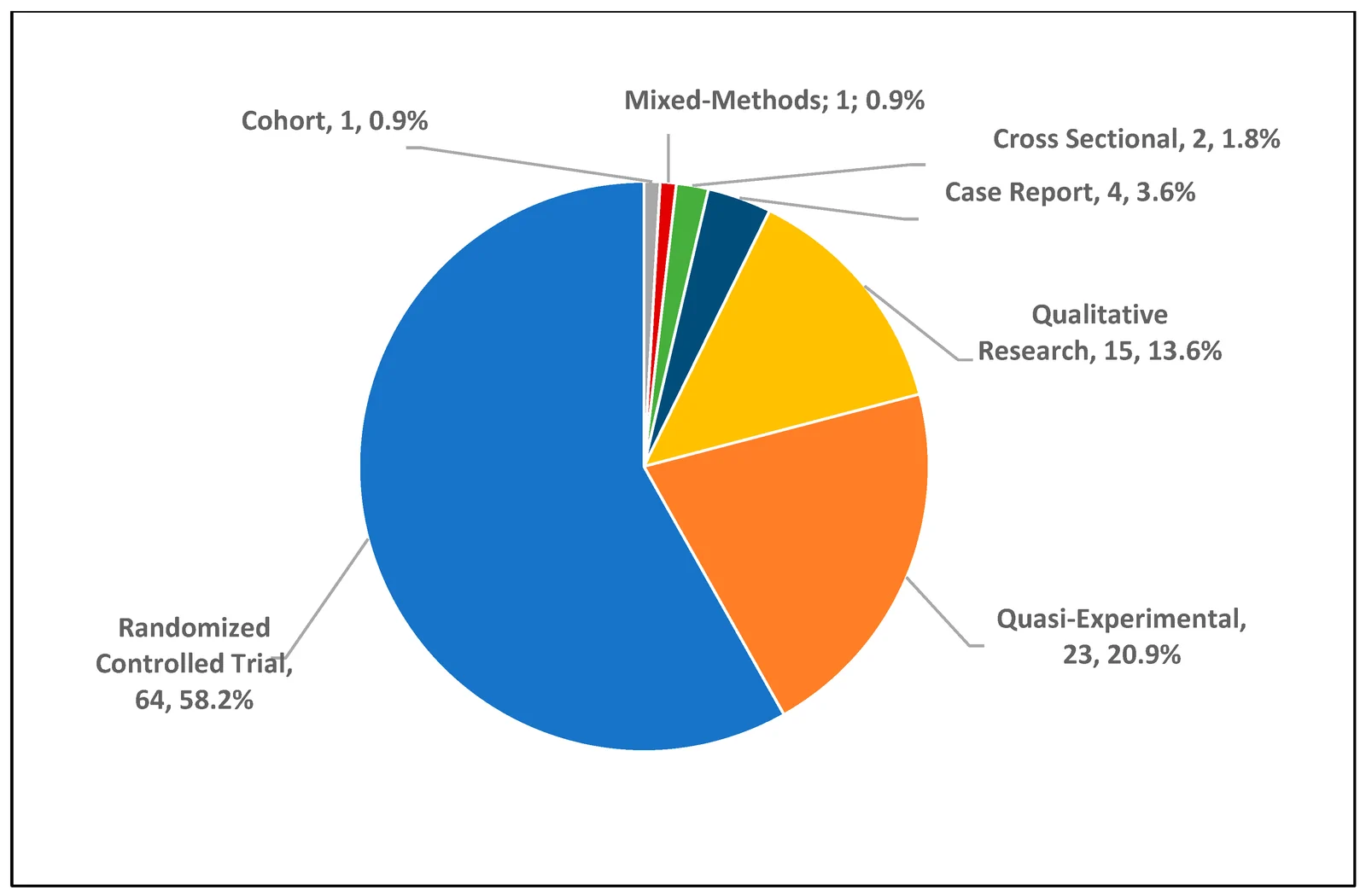
Distribution of study designs among the 110 included studies. Percentages were calculated using the total number of included studies (N = 110) as the denominator.

The 110 included studies employed a range of study designs (see Figure 2). Descriptive study designs were represented by case reports (4/110 studies, 3.6%). Experimental designs included randomized controlled trials (64/110 studies, 58.2%) and quasi-experimental studies (23/110 studies, 20.9%). Observational designs included qualitative research (15/110 studies, 13.6%), cross-sectional research (2/110 studies, 1.8%), mixed-methods studies (1/110 studies, 0.9%), and cohort studies (1/110 studies, 0.9%).

#### 3.1.2. Global Publication Trend on Storytelling and Immunization

**Figure 3:**
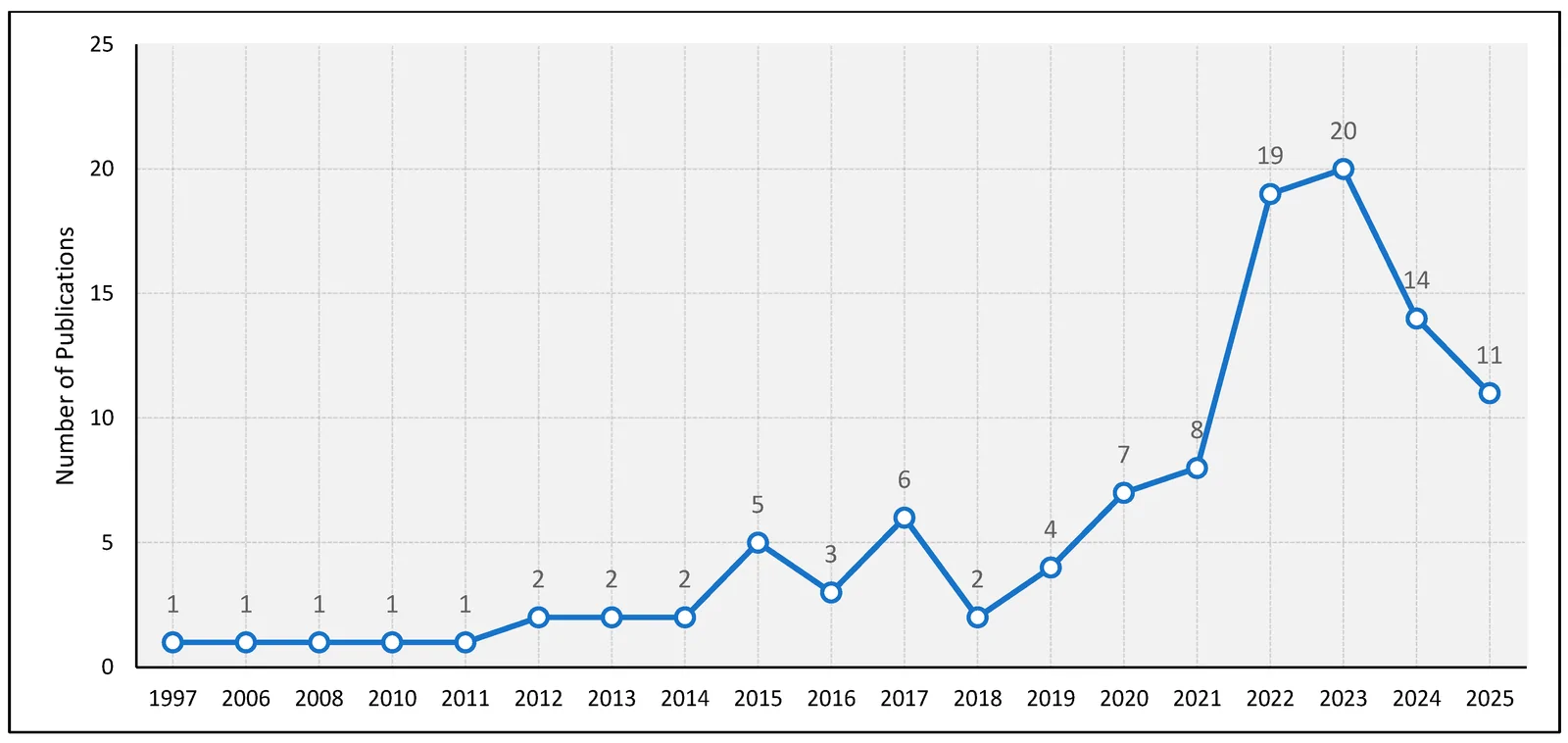
Global publication trends on storytelling and immunization (1997–2025).

Our findings indicate that the connection between storytelling and immunization was first documented in 1997, with noticeable growth from 2015 onward. A rapid increase is observed from 2021 to 2023, followed by a gradual decline in 2024. This decline may be a lag in publications, given that the searches were conducted in March 2025 (see Figure 3).

**Figure 4:**
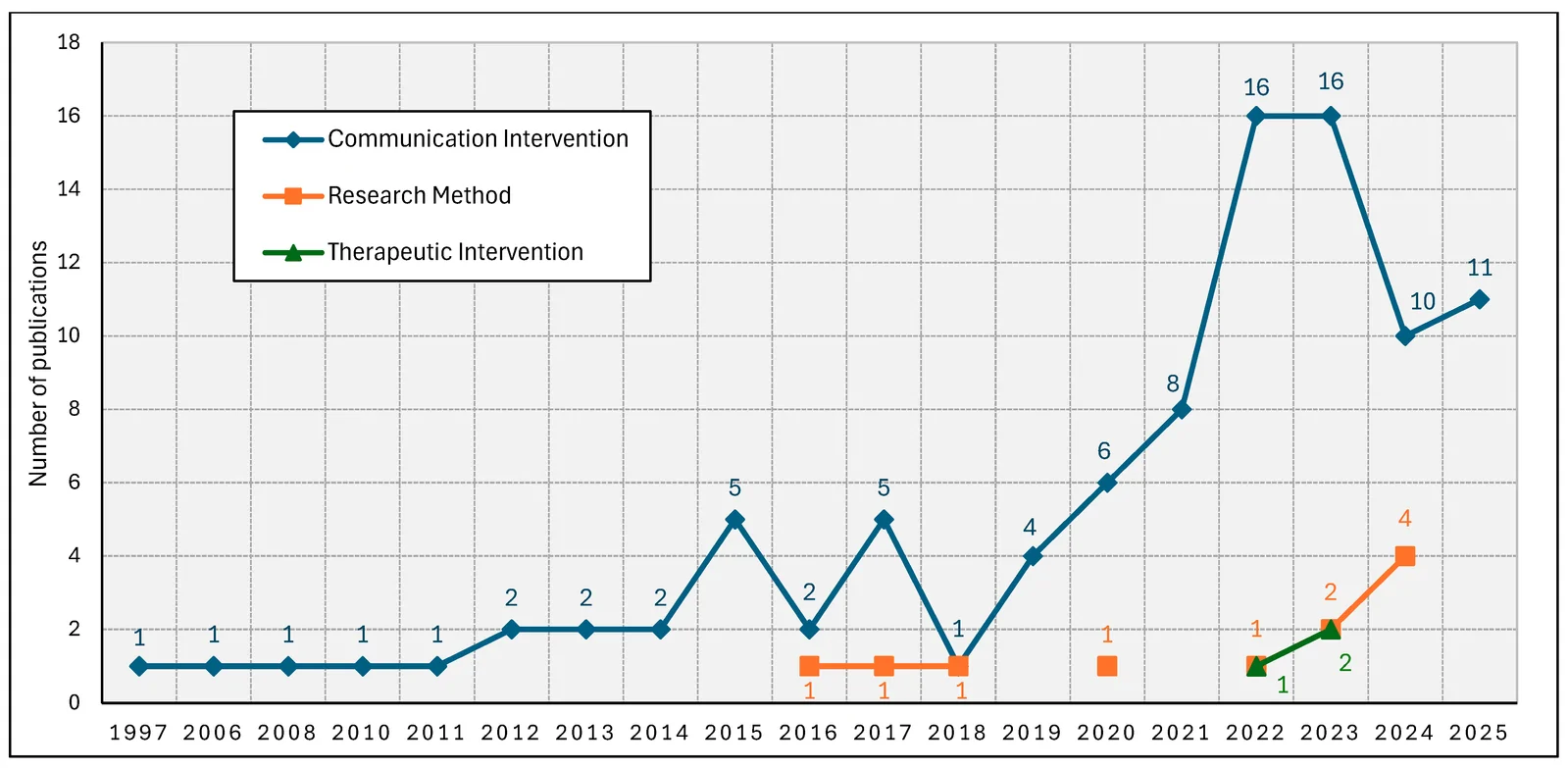
Storytelling application in immunization by year of publication (1997–2025).

Storytelling was consistently used as a communication intervention, with a few studies showing its application in participatory research from 2016 to 2024, suggesting its growing relevance in both vaccination-related health communication and community engagement research. In 2021 and 2022, a few studies reported an additional use of storytelling as a therapeutic approach in immunization (see Figure 4). Its application was also observed at different time points across various vaccine types. Storytelling related to the human papillomavirus (HPV) vaccine was the most consistent across the years in the included studies (47/110 studies), followed by COVID-19 (26/110 studies), the mumps, measles, and rubella (MMR) combination vaccine (10/110 studies), influenza (7/110 studies), childhood vaccines (7/110 studies), general non-specified vaccines (6/110 studies), fictitious vaccine (4/110 studies), hepatitis B vaccine (2/110 studies) and a single study on the respiratory syncytial virus (RSV) vaccine (see Table 1).

#### 3.1.3. Regions

**Figure 5:**
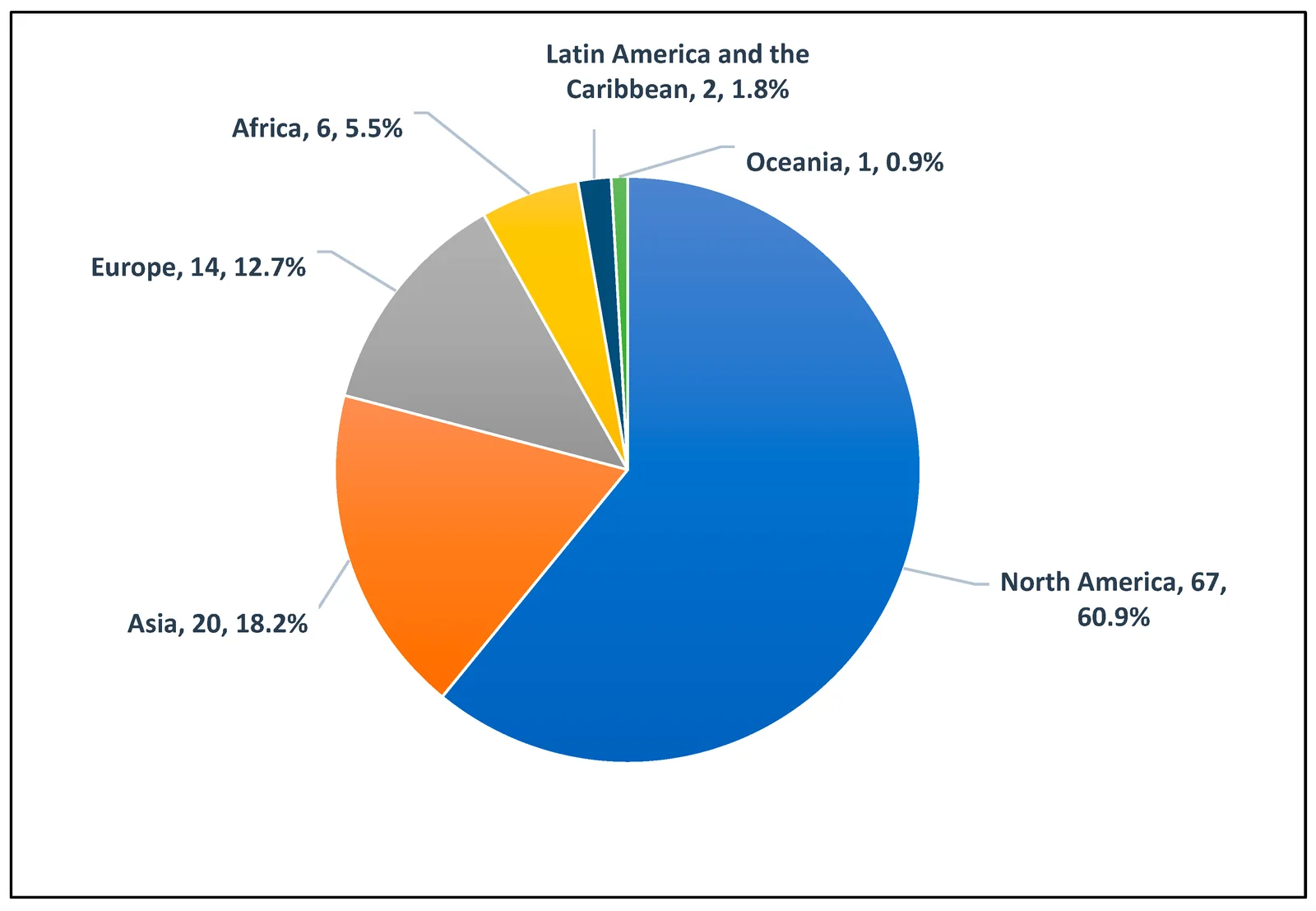
Distribution of the 110 included studies across global regions according to the UN regional classification. Percentages were calculated using 110 as the denominator.

The studies included in the review were conducted across various global regions and were classified using the United Nations regional distribution (see Figure 5). The majority of studies were conducted in North America (67/110 studies, 60.9%), followed by Asia (20/110 studies, 18.2%). The six studies conducted in Africa (6/110 studies, 5.5%) originated from six different countries and employed varied study designs: Ghana (*n* = 1), RCT; Malawi (*n* = 1), qualitative research; Nigeria (*n* = 1), case report; South Africa (*n* = 1), case report; Uganda (*n* = 1), case report; and Zambia (*n* = 1), quasi-experimental.

### 3.2. Component and Application of Storytelling in Immunization Identified from Included Studies

#### 3.2.1. Storytelling Approaches

Of the 110 included studies, the majority used storytelling as a communication intervention (96/110 studies), while a smaller number of studies used storytelling as a research method (12/110 studies) or a therapeutic approach (3/110 studies). Most studies were assigned to a single category, indicating that the classification was largely mutually exclusive. However, one study was assigned to both the communication intervention and research method categories. Categorization was informed by how storytelling was operationalized within each study. Initial classification was conducted by one reviewer and subsequently checked by two additional reviewers for consistency and accuracy. A study was classified under the communication intervention category if storytelling was used as part of an intervention to educate, raise awareness, or influence knowledge, intentions, and behaviors. It was classified under the research category if storytelling was used as a method of data collection. Additionally, storytelling was considered a therapeutic approach when used to reduce stress, pain, or anxiety associated with the vaccine process, or to support healing from stigma and trauma among storytellers (see Table 2).

#### 3.2.2. Storytelling Formats

The included studies made use of a variety of storytelling formats, including oral, audiovisual, and written narratives. Film was the most common storytelling format, reported in 42 out of the 110 studies. We used the Oxford dictionary’s definition of a film being a motion picture that tells a story or conveys information through a sequence of moving images and sound. In this review, films included animation films, documentary films, Hollywood film extracts, and other short testimonial or reenactment videos. Other storytelling formats included narrative text reported in (38/110 studies), storybooks including pictorial storybooks and photo novelas (8/110 studies), social media posts or reels (8/110 studies), audio recordings including radio drama (3/110 studies), illustrations (2/110 studies), oral storytelling (2/110 studies), video games (1/110 studies), live theater/drama (3/110 studies), and songs or music (1/110 studies). The length of visually recorded stories ranged from 1 min to 45 min. The majority of recorded videos were between 1 and 5 min (reported in 17 studies). Fewer videos were longer: 6–10 min (reported in three studies), 11–15 min (reported in four studies), and 30–45 min (reported in two studies). The content of these stories was either testimonial, expository information, re-enactment, fictitious, or a blend of two or more. Some studies reported more than one storytelling format; therefore, the format categories were not mutually exclusive.

#### 3.2.3. Storytelling Delivery Channels

Diverse channels were used in the included studies to share the stories. Online platforms were the most frequently reported dissemination channel, used in 71 of the 110 included studies, followed by school settings (13/110 studies), hospitals or clinics (7/110 studies), laboratories (research units in universities where social experiments are conducted) (5/110 studies), community public spaces (4/10 studies), churches (4/110 studies), homes (3/110 studies), radio (3/110 studies), community based organizations (3/110 studies), libraries (1/110 studies), mail (1/110 studies), and printed posters (1/110 studies). Some studies reported the use of more than one dissemination channel; therefore, channel categories were not mutually exclusive.

#### 3.2.4. Stories Producers and Presenters Identified in the Studies

**Figure 6:**
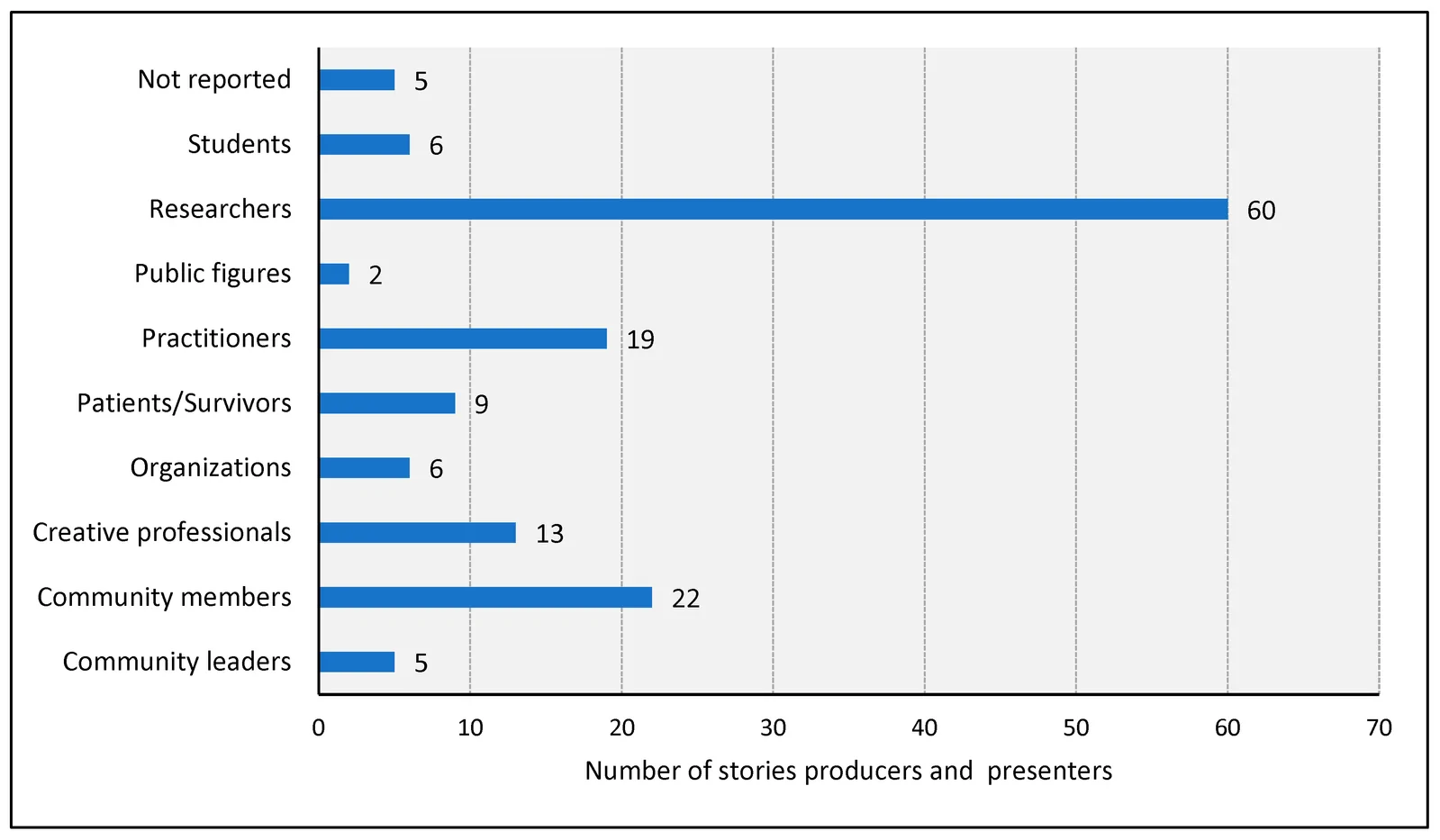
Stories producers and presenters identified across the 110 included studies. Frequencies represent the number of studies reporting each category. Categories were not mutually exclusive because some studies reported multiple producers and presenters.

Given the diversity of individuals involved in the production and presentation of the stories across the included studies, we categorized them into distinct groups for clarity. These are: community leaders (including pastors, business men, and radio station hosts), community members (including parents, pregnant women, and youths and men), creative professionals (including illustrators, writers, singers, film producers, and photographers), organizations (including BBC, UN, UNICEF Ghana, Viamo, Ghana Health Service, Ghana Fact, and the Yale Institute for Global Health, CDC, PVA Africa, religious community organizations, and unspecified NGOs), patients and survivors (including COVID-19, HPV, and hepatitis B patients and survivors), practitioners (including teachers, health authorities, doctors, nurses, CHWs, counselors, and nurses), public figures (peer role models and actress), researchers and students. In some studies, multiple story producers and presenters were reported; therefore, these categories were not mutually exclusive (see Figure 6).

#### 3.2.5. Most Frequently Identified Mediating Factors with Potential to Strengthen the Influence of Storytelling

Some studies examined the mediating factors that may enhance the strength of storytelling. These studies considered the use of the following:

- Hybrid messages (the combination of stories and statistics);
- First-person narrative as opposed to third-person narrative;
- Socio-cultural, linguistic and character representation tailoring to specific audiences;
- Using trusted voices from within the community;
- Loss framing (sometimes called “stories of regret”), as opposed to gain framing; and
- Counterclaim arguments in narrative forms delivered simultaneously with misinformation.

A limited number of studies also explored whether stories might be more effective amongst populations with lower educational levels and/or lower health literacy rates. We cannot comment on whether these additional mediating factors increased or decreased the effectiveness of storytelling, as that is beyond the scope of this review. These identified factors may, nonetheless, be useful for future research.

#### 3.2.6. Key Recurring Theoretical Frameworks Used in the Included Studies

Some theoretical frameworks were repeatedly used to inform the development and implementation of the storytelling approaches as well as the selection of vaccine-related outcomes in the included studies. However, the extent to which these theoretical frameworks were explicitly examined was not consistently or adequately reported. The most frequently used theory is the Narrative Persuasion Theory (17/110 studies). The Narrative Persuasion Theory posits that stories, as opposed to facts alone, are more capable of shaping the attitudes, beliefs, and behavior of people through the reduction in cognitive resistance. It leads to the transportation of individuals into the story world through emotional connection and character identification, making stories memorable and persuasive [51]. The second most commonly used theory in the included studies is the Health Belief Model (11/110 studies). This theory consists of six cognitive constructs, namely perceived susceptibility, perceived severity, perceived benefits, perceived barriers, self-efficacy, and cues to action. The theory explains how individuals perceive these health threats and decide to act based on the value they place on a particular goal and the likelihood that their action toward that goal will be successful in achieving the goal [52,53]. The third most adopted theory in the included studies is the Theory of Planned Behavior (10/110 studies), which suggests that human action is influenced by behavioral intention through the individual’s perceived behavioral control, subjective norm, and attitude towards the behavior [54]. Additionally, there is the Exemplification Theory (7/110 studies), which suggests there is an effect of partial representations of larger phenomena or populations on downstream social perceptions and behavior [55,56]. We equally observed a repeated use of the Elaboration Likelihood Model (6/110 studies), which highlights that persuasion occurs through the central and the peripheral route. The former refers to persuasion that involves deep thinking about the logic of the message, leading to a lasting change, while the latter implies that persuasion is driven by surface cues, leading to a temporal shift [57]. The Transportation Imagery Model (6 of 110 studies) is also frequently observed, which is a theory that explains how individuals are persuaded through an experience of being immersed in the narrative world [58]. Finally, the Narrative Communication Theory (5 of 110 studies) is also known as the narrative paradigm by Walter R. Fischer. He argues that humans are fundamental storytellers who understand and interpret the world through narratives because it resonates on an emotional level and not just through rational and logical arguments [59]. He states that “humans are storytellers; the paradigmatic mode of human decision-making and communication is good reasons which vary in form among communication situations, genres, and media; the production and practice of good reasons is ruled by matters of history, biography, culture, and character along with the kinds of forces identified in the Frentz and Farrell language action paradigm” [60]. Although other theories were adopted in the included studies, they were not used as frequently as those reported above, hence not all presented here.

#### 3.2.7. Vaccine-Related Outcomes Measured

**Figure 7:**
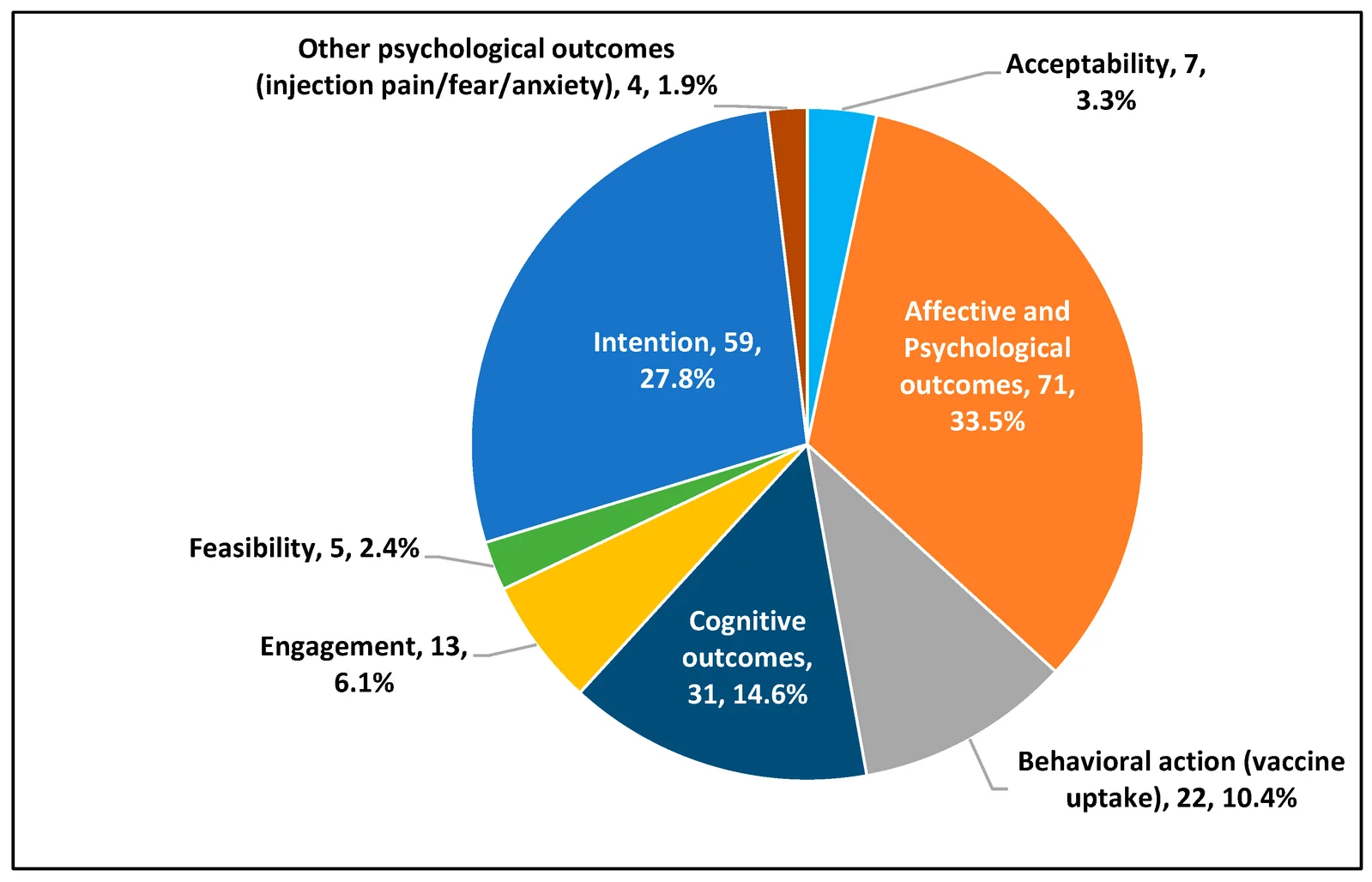
Vaccine-related outcome categories measured across the included studies. Percentages calculated using the total number of vaccine-related outcomes as the denominator (212 outcomes). Categories were not mutually exclusive, as individual studies contributed multiple vaccine-related outcomes.

The classification of vaccine-related outcomes from the included studies was inspired by the WHO’s Behavioral and social drivers of vaccination (BeSD) categorization [61] and the Health Belief Model framework [52]. Outcomes reported across the included studies were grouped into broader vaccine-related outcome categories. Because individual studies frequently assessed multiple vaccine-related outcomes, the outcome categories were not mutually exclusive. Vaccine-related outcome proportions were therefore calculated using 212 total vaccine-related outcomes as the denominator. Affective and psychological outcomes were the most frequently measured outcome category (71/212 outcomes, 33.5%), including attitudes, perceptions, motivation, confidence, trust, information avoidance behavior, and self-efficacy. Intention to vaccinate was the second most frequently measured outcome category (59/212 outcomes, 27.8%), followed by cognitive outcomes (31 outcomes, 14.6%), including knowledge, awareness, recall, and retention. Other outcome categories included vaccine uptake (22/212 outcomes, 10.4%), engagement (13/212 outcomes, 6.1%) including participants’ engagement with the stories, and healthcare workers’ engagement with patients through storytelling, acceptability (7/212 outcomes, 3.3%) the feasibility of the intervention (5/212 outcomes, 2.4%) and other psycho-logical outcomes (4/212 outcomes 1.9%), including fear/anxiety related to injection pain, stigma and trauma healing of the storyteller (see Figure 7).

Vaccine-related outcomes were identified from the aims and methods described in each study. Although not all outcomes listed in Table 3 were eventually measured in the reported studies, mapping these outcomes was important to capture the full scope of vaccine-related outcomes considered in the literature. Of the nine vaccine categories listed in Table 3, intention was discussed across eight vaccine types. Affective and psychological vaccine-related outcomes of storytelling were identified across five vaccine types and cognitive outcomes across five vaccine types. Actual vaccine uptake was discussed mostly across HPV vaccine-related studies. In contrast, only two COVID-19 studies discussed the actual uptake of the vaccines, followed by one MMR study and one study on general vaccines. No actual vaccine uptake was reported for childhood vaccines, hepatitis B vaccine, influenza, and RSV vaccine-related studies. Additionally, engagement was only reported for three vaccine types, and similarly, other psychological outcomes were also reported for only three vaccine types. Finally, acceptability and feasibility were reported in a single study each. Frequencies represent outcomes assigned to each category across the included studies. Some studies contributed multiple outcomes. Therefore, the categories were not mutually exclusive (see Table 3).

#### 3.2.8. Reported Changes in Vaccine-Related Outcomes Following Storytelling Interventions

Out of 62 studies that reported vaccine-related outcomes of storytelling, 50 reported changes in vaccine-related outcomes following exposure to a story. Most reported changes were observed in cognitive outcomes (knowledge, awareness, recall, and retention), affective and psychological outcomes (attitudes, perceptions, confidence, trust, and motivation), and behavioral intentions (intention to vaccinate). In contrast, comparatively fewer studies assessed behavioral outcomes such as actual vaccine uptake. Positive changes were far more common and described in 42 studies, while 8 studies reported negative effects of exposure to a story. These negative vaccine-related outcomes appeared in studies where storytelling conveyed misinformation, conspiracy theories, anti-vaccination messages, negative anecdotes, counterarguments, or stories describing vaccine adverse events. These findings should not be interpreted as evidence of effectiveness because no risk of bias assessment was conducted and this scoping review was not designed to evaluate causal effects. Rather, they provide an overview of vaccine-related outcomes reported in the existing literature and may inform future research. This data may also indicate bias in the literature toward the publication of positive results (see Table 4).

## 4. Discussion

### 4.1. Key Findings

This scoping review included a total of 110 studies that explored the use of storytelling in vaccination contexts. It provides a clear map of the different approaches to storytelling used across vaccine types, geographical regions, and time, including its formats and delivery channels. The expanded body of evidence in this review highlights the potential role for storytelling in vaccine-related communication and engagement. In addition, the use of storytelling as both a research and therapeutic approach in the included studies demonstrates the potential diversity in the application of storytelling in vaccine-related topics. A significant finding was the growing application of storytelling across vaccine types over time and the use of RCTs in storytelling and vaccine-related studies, reflecting an increasing interest in the evaluation of storytelling interventions. However, considering the heterogeneity in study designs, populations, vaccine types, settings, storytelling formats, delivery channels, and outcome measures, together with the absence of a risk-of-bias assessment, the findings should be interpreted with caution. Even so, the evidence mapped in this review indicates growing interest in storytelling as an adaptable and flexible approach for addressing contextual vaccine-related challenges and highlights its potential role in vaccine-related communication and engagement across diverse population groups.

### 4.2. Application of Storytelling in Immunization Across Time and Geographical Location

The findings of this scoping review highlight a growing global interest in the application of storytelling as a communication intervention, participatory research method, and therapeutic approach in immunization. Despite the majority of the evidence being from the USA, this review found a steady growth in the use of storytelling as a communication intervention from 1997 to 2025. While storytelling was consistently used as a communication intervention across the years, its additional use emerged in participatory research from 2016 to 2024, and as a therapeutic approach in 2021 and 2022. This suggests a broader conceptualization of how storytelling is adopted in the field of immunization. Its peak use between 2021 and 2023 was likely influenced by heightened demand for effective vaccine communication and trust-building strategies during the COVID-19 pandemic, a period marked by widespread misinformation and vaccine hesitancy globally [62,63,64].

An important observation is the growing adoption of storytelling across multiple vaccine types, a demonstration of its flexibility and potential usefulness in both pandemic preparation and the introduction of new vaccines [65]. The prominence of HPV-related studies is notable and may reflect longstanding challenges in HPV vaccine uptake, where storytelling has been used to address sensitive social dynamics around gender, sexuality, and parental decision-making, with concerns such as persuasion, deception, and manipulation being noted as ethical concerns [23,66]. The high number of COVID-19 studies similarly reflects the rapid mobilization of communication research in response to the pandemic [67]. While a greater number of the studies included in this review were conducted in high-income countries, only six studies were carried out in Africa. This geographical disparity raises important methodological and ethical questions about the generalizability of existing findings, particularly given that vaccine-preventable disease burdens are often highest in low- and middle-income regions. Because the evidence we have available is from contexts that differ significantly from those settings in low- and middle-income countries where solutions that drive up vaccine coverage are most urgently needed, we need to be particularly careful in interpreting the results. Although storytelling has been consistently regarded as a core African practice, the limited number of studies from Africa included in this review, signals a significant gap in published research on this topic from the continent. This is likely due to limited funding and publication bias, rather than a lack of storytelling practices consistent with the broader literature on factors contributing to publication disparities and a true absence of storytelling practices [68]. Given the long-standing importance of storytelling in many African cultural contexts, the literature is unlikely to have fully captured the extent to which storytelling is used in practice [69]. This highlights an ethical imperative for more investment in local research to enhance adequate representation of African experiences. This would help to close the visibility gap in global research and provide the crucial evidence needed to inform decisions about the use of storytelling to increase vaccine interventions across Africa [70].

### 4.3. Implications of Storytelling for Vaccine-Related Outcomes

The expanded body of evidence in this review suggests that storytelling might have relevance for a range of vaccine-related outcomes reported in the included studies. However, the available evidence was concentrated primarily on knowledge and cognitive outcomes, affective and psychological outcomes, and behavioral intentions, whereas comparatively fewer studies assessed behavioral outcomes such as actual vaccine uptake. Most included studies were experimental designs reflecting an ongoing interest in understanding the relationships between storytelling interventions and vaccine-related outcomes. The inclusion of a great number of experimental studies provided a glimpse of the potential influence of storytelling on vaccine-related outcomes. This body of literature warrants further evaluation of the risk of bias in the included studies and a more rigorous assessment of intervention effectiveness before conclusions can be drawn with confidence.

The diversity of storytelling formats and channels adopted in the included studies demonstrates the flexibility and adaptability of storytelling in relation to different health communication needs. The use of storytelling in included studies as a research method highlights its relevance in building trust and improving engagement in communities experiencing research fatigue while shifting community members from mere passive informers to active contributors. The diversity of people involved in the production and presentation of storytelling further strengthens the value of community participation and collaboration in addressing immunization challenges. Most vaccine-related outcomes were sparsely measured across vaccine types. Actual vaccine uptake was discussed mostly in HPV-related studies. In contrast, only two COVID-19 studies, one MMR study, and one study on general vaccines assessed actual uptake outcomes. No actual vaccine uptake data were collected in any childhood vaccine, hepatitis B, influenza, or RSV-related studies. This imbalance between attitudinal and behavioral outcomes is a notable gap in the current evidence base, given that behavior change is the goal of vaccination communication. It might reflect the logistical difficulty of measuring uptake within experimental designs, though it limits conclusions about the real-world impact of storytelling interventions. Overall, the findings from this review underline the need to consider communication approaches that engage with the emotions of people, as multiple included studies suggested that stories might be more persuasive, engaging, and memorable than purely factual content alone [24,71]. The negative influence of storytelling reported in a couple of studies when used in harmful and misleading narratives demonstrated that its persuasive power could work in both directions. These findings suggest that emotionally engaging approaches including storytelling warrant further evaluation within vaccination communication strategies while taking into account the ethical risks associated with storytelling. Some of these risks include the potential for emotional overreach, whereby highly affective stories could disproportionately influence perceptions of risk, anecdotal distortion, in which individual experiences are generalized beyond their evidentiary value, and the selective presentation of information, which might obscure the balance of benefits and risks.

### 4.4. Mediating Factors and Theories Underpinning the Value of Storytelling

A number of mediating factors underpinning the development of storytelling interventions might determine how well the intervention works. More research is needed to assess whether these mediating factors affect vaccine outcomes. The variety of theoretical frameworks informing the storytelling interventions included in this review suggests that theory-informed storytelling might be relevant to a range of vaccine-related outcomes, although this review cannot determine the effectiveness of such interventions.

### 4.5. Implications for Future Research

An effectiveness review of the available intervention research on storytelling and vaccine outcomes is worth exploring. Gaps in the available evidence also suggest that more primary research is needed on the impact of storytelling in vaccine-related outcomes, especially the long-term impact of storytelling translating to real vaccination behaviors. Further research is needed on storytelling’s potential in reversing the negative effect of misinformation given the constant exposure of the general public to anti-vaccination content. More research is also needed on the feasibility of storytelling in immunization, including dosage and cost-effectiveness. Finally, more research on the use of storytelling in immunization in Africa are needed.

### 4.6. Limitations

While we set out to comprehensively map the existing evidence, we excluded studies for which there was no full text available online; hence, we may have inadvertently missed some studies. Secondly, although no language restriction was applied, language and indexing bias might still have potentially influenced study inclusion. Additionally, the included studies were highly heterogeneous in terms of study design, population, vaccine types, and settings, limiting comparability and the possibility to draw consistent or generalizable conclusions. The absence of risk of bias assessment constitutes a limitation of this scoping review. While this approach is consistent with standard scoping review methodology and reflects our aim of providing a high-level overview of the available literature, it limits the ability to assess the quality and reliability of the findings. This review did not explore the application of theoretical frameworks and theories of change within the available evidence base. Further analysis in a full review would enable more detailed exploration of the mechanisms at play when storytelling is used. The results should therefore be interpreted with caution. In-depth analysis, assessment of bias, and synthesis of the effectiveness studies would add further to this work. Additionally, most of the studies included in this review were conducted in high-income countries, limiting the generalizability of the findings to other regions such as Africa. Furthermore, most included studies measured only proximal vaccine-related outcomes, such as knowledge, awareness, recall, attitudes, perceptions, confidence, trust, and vaccination intention. Very few studies measured the actual uptake of vaccines. This constitutes an important limitation of the extent to which storytelling interventions translate to change in vaccine behavior. Lastly, it is important to note that, given the nature of this review, no inference can be made regarding the effectiveness of storytelling.

## 5. Conclusions

This scoping review maps a broad and growing body of evidence on the use of storytelling in vaccination. Our findings indicate that storytelling has been applied across a wide range of theoretical frameworks, populations, and contexts, with included studies reporting outcomes related to knowledge, attitudes, intentions, confidence, engagement, and, in very few cases, vaccine uptake. A wide variety of formats and channels have been applied, reflecting the flexibility of storytelling across different populations and contexts. However, significant evidence gaps remain, particularly in low- and middle-income regions, for several vaccine types, and with respect to actual vaccine uptake outcomes. Future research should prioritize building a more geographically representative evidence base, exploring longer-term vaccine-related outcomes, and examining the conditions under which storytelling might be most appropriate and ethically implemented, given its reported capacity to both promote and undermine vaccination. To conclude, while storytelling appears to be a widely used communication approach in vaccination, further analysis of the available evidence, as well as additional evaluation in contexts where vaccination is most urgent, is needed to determine its effectiveness, optimal design, ethical use, and long-term behavioral impact.

## Figures and Tables

**Table 1:** Distribution of storytelling applications across vaccine types in the 110 included studies (1997–2025).

Publication Year	Vaccine Types									
	Childhood	COVID-19	Fictitious	General	Hepatitis B	HPV	Influenza	MMR	RSV	Total
1997				1						1
2006	1									1
2008						1				1
2010						1				1
2011			1							1
2012						1	1			2
2013			1		1					2
2014						1		1		2
2015			1			4				5
2016						3				3
2017						5	1			6
2018						1		1		2
2019						4				4
2020	1				1	4	1			7
2021		2				4	1	1		8
2022	2	6				6	1	4		19
2023	1	7	1	3		6		2		20
2024	1	6		2		3	1	1		14
2025	1	5				3	1		1	11
Total Number of Studies	7	26	4	6	2	47	7	10	1	110

**Table 2:** Operational definitions, coding criteria, and illustrative examples for the categorization of storytelling approaches in the included studies.

Category	Definition	Coding Criteria	Examples
Communication intervention	Communication intervention in health is defined as the strategic use of communication techniques to influence, engage, empower, and support individuals or populations for the promotion of healthy behaviors, mitigation of risks, or improvement of health outcomes [41].	Studies were classified under this category when storytelling was used primarily to engage audiences, raise awareness, transfer knowledge, persuade or influence vaccination-related attitudes, intentions, decisions, or behaviors.	Frett et al. (2016) developed and evaluated culturally tailored Haitian Kreyòl storytelling videos on HPV vaccination and cervical cancer screening [42]. The storytelling intervention aimed to address barriers to prevention and improvement of knowledge and uptake among Haitian women in South Florida. Bufalini et al. (2024) used a video education intervention featuring a cancer survivor narrative to influence parents’ attitudes toward and intentions to initiate HPV vaccination among unvaccinated males aged 9–17 [23].
Research method	Storytelling as a research method places participants at the center of inquiry by collecting, eliciting, analyzing, and presenting individuals’ live stories in their own words and from their own perspectives. As a qualitative research approach, storytelling differs from other qualitative methods in that stories are not merely a source of data but are in themselves the phenomenon under study. This approach preserves the narrative structure of lived experiences, including core elements such as setting, characters, plot, narrative style, and theme [43,44,45].	Studies were classified under this category when storytelling was used as a data collection method.	A study by Harries et al. (2024) encouraged participants to use their mobile smartphones to document their lived experiences related to HIV treatment and care during COVID-19 through photos, videos, voice notes, and text messages [46]. Topics were introduced via Telegram every 2 to 4 weeks, covering areas like HIV treatment, mental health, and the COVID-19 vaccine. Participants received training on using digital tools and monthly data/airtime. The intervention aimed at enabling remote engagement, depth, and reflection beyond traditional qualitative interviews. Gerhardt et al. (2024) used an Ethnographic audiovisual documentary method to construct the social presence of the quilombola community of Morro Alto in political decision-making environments [47]. The developed scripts contained three main themes: the pandemic scenario and its impacts; quilombola identity and recognition; and vaccination. In production, images were captured through interviews, footage of the territory, aerial and photographic images, and reports on the creation of lists of quilombola residents to be vaccinated. The pre-constructed script was adapted through interaction between the camera and interviewees, shaped by expressions of the body-territory, with engagement and involvement of the team in offering a new narrative from the perspective of those who have always been invisible and silenced.
Therapeutic approach	Therapeutic interventions are all activities, treatments, or approaches designed to improve an individual’s physical, psychological, emotional, social, or functional well-being and to alleviate symptoms, distress, or impairment [48].	Storytelling was considered a therapeutic approach when used to reduce stress, pain, or anxiety associated with the vaccine process, or to support healing from stigma and trauma among storytellers.	Dyah Listyarini et al. (2022) used counseling with the storytelling method on the anxiety of school learners to receive COVID-19 vaccination in primary school [49]. Sarah et al. (2023) narrated a pictorial story in their study before the immunization procedure [50]. The story contained information about anxiety related to immunization, coping strategies, and distraction techniques. It was presented in a book format, with pictures and text depicting a child’s visit to the immunization clinic step by step, ending with the child going home happy. The story was developed in a culturally appropriate format and translated into the local language.

**Table 3:** Distribution of vaccine-related outcome categories across vaccine types in the included studies. Frequencies represent outcomes assigned to each category. Categories were not mutually exclusive because some studies contributed multiple outcomes.

Vaccine-Related Outcomes of Storytelling	Vaccine Types									
	Childhood	COVID-19	Fictitious	General	Hepatitis B	HPV	Influenza	MMR	RSV	Total
Acceptability						1				1
Affective and Psychological outcomes	3	13				15	4	3		38
Behavioral action (vaccine uptake)		2		1		6		1		10
Cognitive outcomes	1	3		4	1	7				16
Engagement		2				4		1		7
Feasibility						1				1
Intention	2	6	4		1	15	3	4	1	36
Other psychological outcomes		1		1				1		3
Grand Total	6	26	4	6	2	46	7	10	1	113

**Table 4:** Summary of vaccine-related outcomes reported following storytelling interventions.

Vaccine-Related Outcomes Measured	Number of Studies	Change in Outcomes	No Change in Outcomes
Knowledge and cognitive outcomes (knowledge/awareness/recall/retention)	14	11	3
Affective and psychological outcomes (attitudes/perceptions/motivation/confidence/trust)	15	11	4
Behavioral intention (intention to vaccinate)	13	10	3
Behavioral action (vaccine uptake)	7	6	1
Feasibility	2	1	1
Acceptability	2	2	0
Other psychosocial/physical outcomes (response to injection pain/emotional reactions)	3	3	0
Engagement	6	6	0
Total Count	62	50	12

## Data Availability

The data supporting the findings in this review will be made available from the corresponding authors upon request.

## Supplementary Materials

The following supporting information can be downloaded at: https://www.mdpi.com/article/10.3390/vaccines14080665/s1, Supplementary File S1: Search strategy; Supplementary File S2: Scoping review search records; Supplementary File S3: Data extraction form.

## Abbreviations

The following abbreviations are used in this manuscript:

JBI	Joanna Briggs Institute
RoB 2	Cochrane Risk of Bias 2 tool
PRISMA-ScR	Preferred Reporting Items for Systematic Reviews and Meta-Analyses—Extension for Scoping Reviews
RCTs	Randomized Controlled Trials
HPV	Human Papillomavirus
RSV	Respiratory Syncytial Virus
MMR	Measles, Mumps, and Rubella Vaccine
