# Supplementary material for: Exploring the Use of Storytelling in Vaccination: A Scoping Review
Source: Vaccines (Basel). 2026 Jul 30;14(8):665. doi: 10.3390/vaccines14080665 (PMC13517324; doi:10.3390/vaccines14080665)
Supplement: Supplementary file 1 [file vaccines-14-00665-s001.zip › vaccines-4348128-supplementary.pdf]

vaccines

MDPI

Supplementary materials

Supplementary File S1: Search Strategy

Population: Public, citizens, health practitioners, health authorities, health researchers

Concept: Storytelling

Context: Immunization, vaccination

Concepts/synonyms

Population	Concepts	Context
Parents, adults, practitioners Citizens, the public, people, health workers, decision-makers, policymakers, researchers, clinicians, nurses, community health workers, doctors, women, mothers, caregivers, patients.	Storytelling, story, lived experience, shared knowledge shared experience, narration, narrative anecdote, testimonial, documentary film, film, visual arts, graphic, animation.	Immunization, vaccination, routine childhood immunization, Expanded Programme on Immunization, EPI, Vaccines.

Date	Database	Query	Record
03/03/2025	PubMed	Search: **(Storytelling[Title/Abstract] OR stories[Title/Abstract] OR story[Title/Abstract] OR "lived experience"[Title/Abstract] OR "lived experiences"[Title/Abstract] OR "shared knowledge"[Title/Abstract] OR "shared experience"[Title/Abstract] OR "shared experiences"[Title/Abstract] OR narration[Title/Abstract] OR narrative[Title/Abstract] OR narratives[Title/Abstract] OR anecdote[Title/Abstract] OR anecdotes[Title/Abstract] OR testimonial[Title/Abstract] OR testimonials[Title/Abstract] OR "documentary film"[Title/Abstract] OR film[Title/Abstract] OR films[Title/Abstract] OR "visual arts"[Title/Abstract] OR graphic[Title/Abstract] OR graphics[Title/Abstract] OR animation[Title/Abstract] OR animations[Title/Abstract]) AND (Immunization[Title/Abstract] OR "routine childhood immunization"[Title/Abstract] OR "Expanded Programme on Immunization"[Title/Abstract] OR EPI[Title/Abstract] OR vaccination[Title/Abstract] OR "childhood vaccination"[Title/Abstract] OR Vaccine[Title/Abstract] OR vaccines[Title/Abstract])** Filters: **Humans**	2,223

Supplementary File S2: Scoping review Search Records

Database	Dates	Search terms	
PubMed	14//04/2025	**Search: (Storytelling[Title/Abstract] OR stories[Title/Abstract] OR story[Title/Abstract] OR "lived experience"[Title/Abstract] OR "lived experiences"[Title/Abstract] OR "shared knowledge"[Title/Abstract] OR "shared experience"[Title/Abstract] OR "shared experiences"[Title/Abstract] OR narration[Title/Abstract] OR narrative[Title/Abstract] OR narratives[Title/Abstract] OR anecdote[Title/Abstract] OR anecdotes[Title/Abstract] OR testimonial[Title/Abstract] OR testimonials[Title/Abstract] OR "documentary film"[Title/Abstract] OR film[Title/Abstract] OR films[Title/Abstract] OR "visual arts"[Title/Abstract] OR graphic[Title/Abstract] OR graphics[Title/Abstract] OR animation[Title/Abstract] OR animations[Title/Abstract]) AND (Immunization[Title/Abstract] OR "routine childhood immunization"[Title/Abstract] OR "Expanded Programme on Immunization"[Title/Abstract] OR EPI[Title/Abstract] OR vaccination[Title/Abstract] OR "childhood vaccination"[Title/Abstract] OR Vaccine[Title/Abstract] OR vaccines[Title/Abstract]) Filters: Humans**	2232
Scopus	14/04/2025	**(TITLE(Story* OR "lived experience" OR narrat* OR "shared knowledge" OR "shared experience" OR testimonial OR anecdote OR anecdotal OR**	743

 https://doi.org/10.3390/xxxxx

		**testimonial OR "documentary film" OR film OR "visual arts" OR graphic OR animation) AND TITLE(vaccin* OR "childhood vaccin*" OR immuniz* OR "routine immunization" OR "expanded programme on immunization" OR epi OR "routine childhood immunization"))**	
EbscoHost	17/04/2025	**TI (Story* OR "lived experience" OR narrat* OR "shared knowledge" OR "shared experience" OR testimonial OR anecdote OR anecdotal OR testimonial OR "documentary film" OR film OR "visual arts" OR graphic OR animation) AND TI (vaccin* OR "childhood vaccin*" OR immuniz* OR "routine immunization" OR "expanded programme on immunization" OR epi OR "routine childhood immunization")<br>Medline(529); Academic search premiere (318) CINAHL(187); APA PsycInfo(74); Africa-wide information (33) Communication and mass media complete (33); socINDEX with full text (20)**	**817**
Web of Science core collections	18/04/2025	**Story* OR "lived experience" OR narrat* OR "shared knowledge" OR "shared experience" OR testimonial OR anecdote OR anecdotal OR testimonial OR "documentary film" OR film OR "visual arts" OR graphic OR animation (Title) and vaccin* OR "childhood vaccin*" OR immuniz* OR "routine immunization" OR "expanded programme on immunization" OR epi OR "routine childhood immunization" (Title)**	621
SciElo via Web of Science		**Story* OR "lived experience" OR narrat* OR "shared knowledge" OR "shared experience" OR testimonial OR anecdote OR anecdotal OR testimonial OR "documentary film" OR film OR "visual arts" OR graphic OR animation (Title) and vaccin* OR "childhood vaccin*" OR immuniz* OR "routine immunization" OR "expanded programme on immunization" OR epi OR "routine childhood immunization" (Title)**	11
Cochrane	22/04/2025	**Trials matching Storytelling OR stories OR story OR "lived experience" OR "lived experiences" OR "shared knowledge" OR "shared experience" OR "shared experiences" OR narration OR narrative OR narratives OR anecdote OR anecdotes OR testimonial OR testimonials OR "documentary film" OR film OR films OR "visual arts" OR graphic OR graphics OR animation OR animations in Record Title AND Immunization OR "routine childhood immunization" OR "Expanded Programme on Immunization" OR EPI OR vaccination OR "childhood vaccination" OR Vaccine OR vaccines in Record Title - (Word variations have been searched)**	**68**
Grey Literature			
Primo	18/04/2025	**Title contains Storytelling OR stories OR story OR "lived experience" OR "lived experiences" OR "shared knowledge" OR "shared experience" OR "shared experiences" OR narration OR narrative OR narratives OR anecdote OR anecdotes OR testimonial OR testimonials OR "documentary film" OR film OR films OR "visual arts" OR graphic OR graphics OR animation OR animations<br>AND Title contains Immunization OR "routine childhood immunization" OR "Expanded Programme on Immunization" OR EPI OR vaccination OR "childhood vaccination" OR Vaccine OR vaccines**	1638
Social Science Research Network		**No results (0)**	
Open Grey		**No longer available**	
Google scholar		**allintitle: (Storytelling OR stories OR story OR "lived experience" OR "lived experiences" OR "shared knowledge" OR "shared experience" OR "shared experiences" OR narration OR narrative OR narratives OR**	101

		**anecdote OR anecdotes OR testimonial OR testimonials OR "documentary film" OR film OR films OR "visual arts" OR graphic OR graphics OR animation OR animations) AND (Immunization OR "routine childhood immunization" OR "Expanded Programme on Immunization" OR EPI OR vaccination OR "childhood vaccination" OR Vaccine OR vaccines)**	
Google		**allintitle: "lived experiences" ǀ "shared knowledge" ǀ "shared experience" ǀ "shared experiences" ǀ narration ǀ narrative ǀ narratives ǀ anecdote ǀ anecdotes ǀ testimonial ǀ testimonials ǀ "documentary film" ǀ film ǀ films ǀ "visual arts" ǀ graphic ǀ graphics ǀ animation ǀ animations AND Immunization**<br><br>**Manual retrieval of additional resources from organizational websites.**	5
**Total Hits to be exported for screening**			**6236**

Supplementary File S3: Data extraction form

Indicator	Description
**Study ID**	Unique identifier for each study
**Title**	Full title of the study
**Lead authors contact details**	Email address or other contact information of the lead author
**Country of study**	Country where the study was conducted
**Aim/Research question**	Main objective or question the study aimed to answer
**Study design**	Type of research methodology employed
**Start date**	Date the study began
**End date**	Date the study concluded
**Study funding sources**	Organization(s) that funded the study
**Possible conflicts of interest**	Any potential biases identified among study authors
**Population description**	Characteristics of the study participants
**Inclusion criteria**	Factors that made participants eligible for the study
**Exclusion criteria**	Factors that prevented individuals from participating
**Method of recruitment**	How participants were invited to participate
**Total number of participants**	Number of individuals included in the study
**Baseline population characteristics**	Characteristics of the study population at baseline
**Interventions**	Description of the intervention/s
**Tools/Questionnaires used**	Specify the tools, measures or questionnaires used in data collection
**Table preview (if applicable)**	Include a brief overview of the table(s) used to present results (e.g., themes, participant characteristics)
**Results**	Key findings of the study in a concise format
**Main results themes**	Broader categories summarizing the key findings
**Strengths**	Positive aspects of the study design and methodology
**Limitations**	Potential weaknesses or limitations of the study
**Conclusion/Key messages**	Main takeaway points from the study
